# Impacts of landscape visual elements on the physiological and psychological responses of recreational visitors in urban pocket parks

**DOI:** 10.3389/fpubh.2026.1868221

**Published:** 2026-09-08

**Authors:** Yabing Huang, Jie Tian, Hongming Wang, Shiyang Xu, Kunxuan Xiao, Jingru Chen

**Affiliations:** 1 College of Fine Arts, Minjiang University, Fuzhou, China; 2 College of Architecture and Landscape, University of Sheffield, Sheffield, United Kingdom; 3 Beijing Forestry University, Beijing, China; 4 College of Fine Arts and Design, Jimei University, Xiamen, China

**Keywords:** environmental preference, physiological response, pocket parks, restorative evaluation, visual elements

## Abstract

Pocket parks are small and accessible urban green spaces that may support short-term restorative experiences in high-density urban environments. However, evidence remains limited regarding how specific visual landscape characteristics are associated with psychological and physiological responses. This study quantified 11 visual landscape indicators at 30 observation points in 10 pocket parks in Fuzhou, China, using panoramic imagery and semantic segmentation. Repeated observations were collected from 50 university students during field exposure, including measures of environmental preference, perceived restorativeness, heart rate variability, and skin conductance. Multiple linear regression models were used as the primary analysis, with linear mixed-effects models conducted as a supplementary sensitivity analysis to account for the repeated-measures structure. The primary analyses indicated that higher proportions of trees, shrubs, lawns, and distant mountain views were generally associated with higher ratings of environmental preference and perceived restorativeness, whereas higher proportions of buildings, hard-paved surfaces, and fences, together with greater spatial enclosure, were generally associated with less favorable psychological evaluations. Selected physiological indicators, including heart rate, pNN50, LF/HF, and skin conductance level, were also associated with visual landscape characteristics. Stratified analyses further suggested that these associations varied across recreational space types, with vegetation-related characteristics showing particularly notable associations in architectural/structural spaces. The supplementary mixed-effects analyses indicated that some context-specific associations remained evident after accounting for repeated observations, particularly the positive associations between shrub proportion and environmental and physiological responses in architectural/structural spaces. Taken together, these findings suggest that the relationships between visual landscape characteristics and psychological and physiological responses may be context-dependent. The findings provide evidence that vegetation visibility, visual openness, and the configuration of landscape elements may inform health-supportive and restoration-oriented pocket park design. However, the observed relationships should be interpreted as short-term associations specific to the participant sample, study sites, and field conditions examined.

## 1 Introduction

Rapid urbanization has reduced the availability of natural green spaces and intensified environmental pressures that may adversely affect residents’ quality of life and well-being (1). Urban parks and other green spaces are therefore increasingly incorporated into urban planning as accessible settings for recreation, contact with nature, and restorative experiences. The quantity and accessibility of these spaces are associated with patterns of park use and a range of health-related experiences (2). Many cities have consequently sought to improve the provision, accessibility, and management of urban green spaces as part of broader efforts to enhance urban environmental quality and human well-being (3).

However, expanding park provision while keeping access distances short is particularly difficult in high-density urban areas, where land availability and financial resources are constrained (4). In central districts, these constraints limit opportunities for developing large new parks and contribute to the increasing provision of smaller and more spatially fragmented green spaces (4–8).

Against this background, pocket parks have emerged as a flexible form of urban green infrastructure that can be integrated into streets, alleys, and other residual or fragmented urban spaces. Their small scale, dispersed distribution, and accessibility make them potentially valuable settings for everyday recreation and short-term restorative experiences in high-density neighborhoods (9, 10). Research on pocket parks has expanded across multiple disciplines (11–13). However, many existing studies have relied primarily on subjective landscape evaluations and questionnaire-based assessments (14, 15). A fuller understanding of visitors’ responses therefore requires consideration of both perceived evaluations and short-term physiological responses to specific environmental characteristics.

Stress Recovery Theory (SRT) and Attention Restoration Theory (ART) provide complementary explanations for how exposure to natural environments may be related to psychological and physiological responses. SRT proposes that visual contact with natural environments may facilitate recovery from psychophysiological stress by reducing physiological arousal and supporting more positive affective states (16). ART suggests that natural settings may help restore depleted attentional resources through qualities such as fascination, extent, and compatibility (17). Together, these theories indicate that the visual composition of green spaces may be relevant to both perceived restorativeness and short-term physiological responses.

Recent evidence further suggests that the effects of urban nature are not solely determined by the presence of green space, but also by the composition, structure, and visual characteristics of landscape elements. A recent meta-analysis identified a generalized dose–response relationship between urban greenness and mental health, indicating that eye-level greenness and visible vegetation density are significantly associated with psychological restoration and mental well-being (18). Moreover, emerging studies have demonstrated that visual exposure to green infrastructure can influence both subjective evaluations and physiological responses, including emotional states, autonomic regulation, and stress-related indicators (19, 20). These findings suggest that specific visual landscape characteristics, such as tree cover, shrub density, lawn proportion, sky visibility, and spatial enclosure, may contribute differently to restorative experiences and physiological responses.

Landscape assessment has increasingly combined objective environmental measures with subjective evaluations and physiological responses (21–25). However, this integrated approach remains comparatively limited in pocket park research, where questionnaire-based assessments are still commonly used. Consequently, it remains unclear which specific visual landscape characteristics are most consistently associated with environmental preference, perceived restorativeness, and short-term physiological responses.

Accordingly, this study quantified visual landscape characteristics at 30 observation points in 10 pocket parks in Fuzhou, China, and examined their associations with university students’ environmental preference, perceived restorativeness, and selected short-term physiological responses. Specifically, the study aimed to identify which characteristics of vegetation, the built environment, and spatial configuration were associated with these psychological and physiological outcomes. The findings may provide preliminary, context-specific insights for pocket park design.

## 2 Materials and methods

### 2.1 Study area and selection of observation points

Fuzhou has developed an extensive network of urban parks and small neighborhood green spaces under its “City of Thousand Parks” initiative (26). The study was conducted in Gulou District, a densely populated central district of Fuzhou (27). The sampling frame included pocket parks and comparable small recreational green spaces, including linear parks and street-level green spaces, with areas ranging from 400 to 8,000 m^2^.

**Figure 1:**
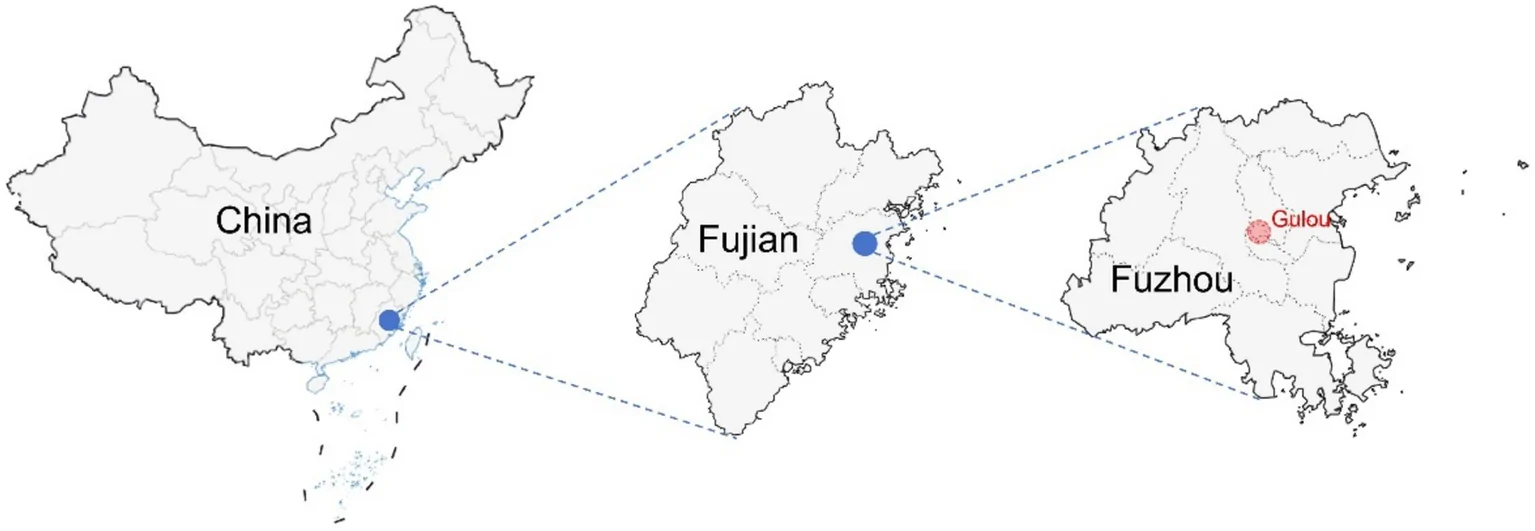
Location of Gulou District, Fuzhou, China.

**Figure 2:**
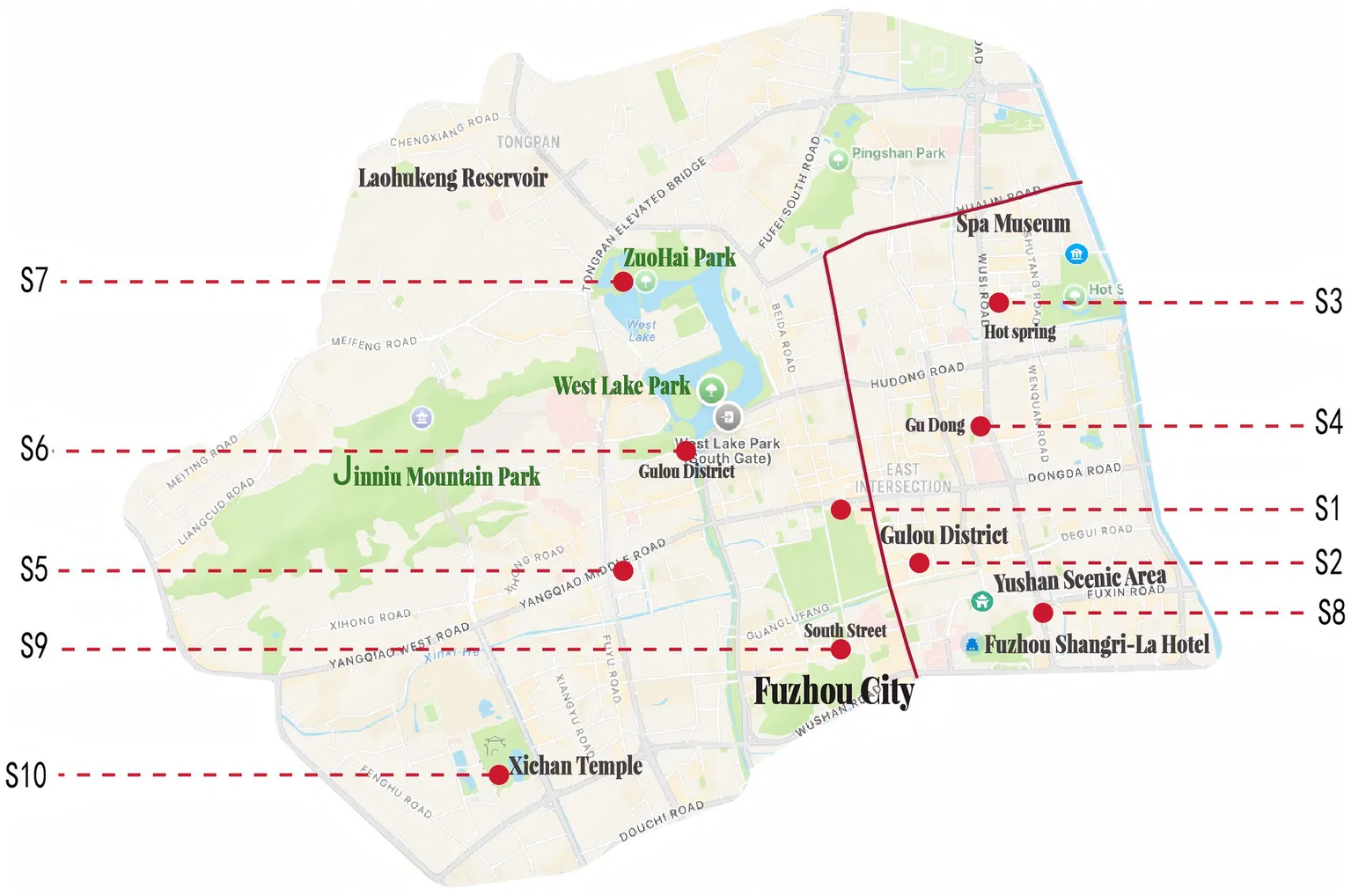
Locations of the ten study sites in Gulou District.

Candidate sites were identified using the *Fuzhou Urban Green Space System Plan* (2016–2020) and satellite imagery were subsequently verified in the field. Ten sites were selected to capture variation in geographic setting, surrounding context, and visual landscape characteristics. Their locations are shown in Figures 1, 2.

Five landscape architecture experts visited the ten study sites between 7 and 10 April 2024. Drawing on field observations, satellite imagery, and group discussion, they selected three observation points within each site according to representativeness, feasibility, and visual diversity. The final sample comprised 30 observation points.

**Figure 3:**
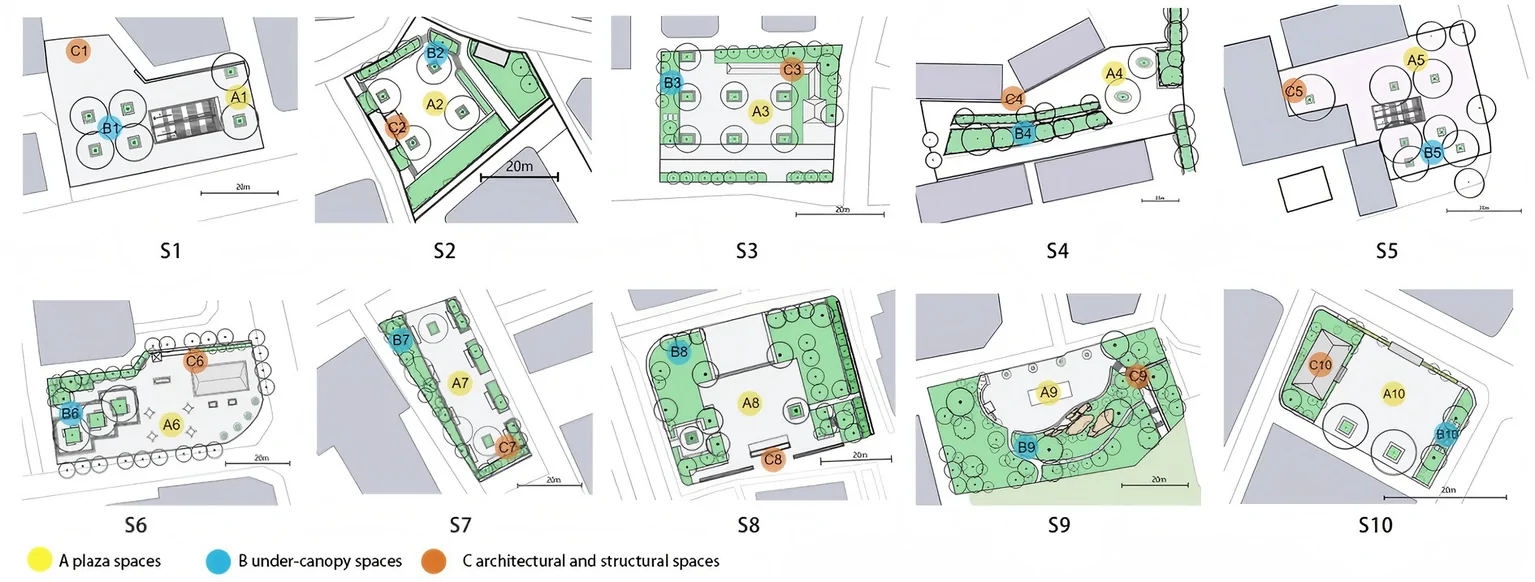
Distribution and classification of the 30 observation points.

To facilitate comparisons among visually distinct environmental settings, the observation points were classified into three recreational space types based on their dominant spatial characteristics and existing site conditions. Type A (plaza spaces) consisted of relatively open areas dominated by hard-paved surfaces and characterized by high visual openness. Type B (under-canopy spaces) referred to resting areas located beneath tree canopies, characterized by substantial vegetation coverage and shaded conditions. Type C (architectural and structural spaces) comprised semi-enclosed areas formed by buildings, pavilions, walls, or other structures, where built-environment features were visually prominent. Each spatial type included ten observation points. The spatial distribution of the observation points within the parks is shown in Figure 3.

### 2.2 Measures and experimental procedures

#### 2.2.1 Visual landscape measures

Eleven visual landscape indicators were derived from the panoramic images and organized into three domains. Vegetation indicators included the green view index and tree, shrub, and lawn proportions. Built-environment indicators included the proportions of structures, buildings, hard pavement, and fences. Spatial indicators included the proportions of visible sky and distant mountains, together with spatial enclosure. The green view index represented the proportion of image pixels classified as vegetation, while spatial enclosure represented the proportion of the visible field occupied by vegetated and built elements.

**Figure 4:**
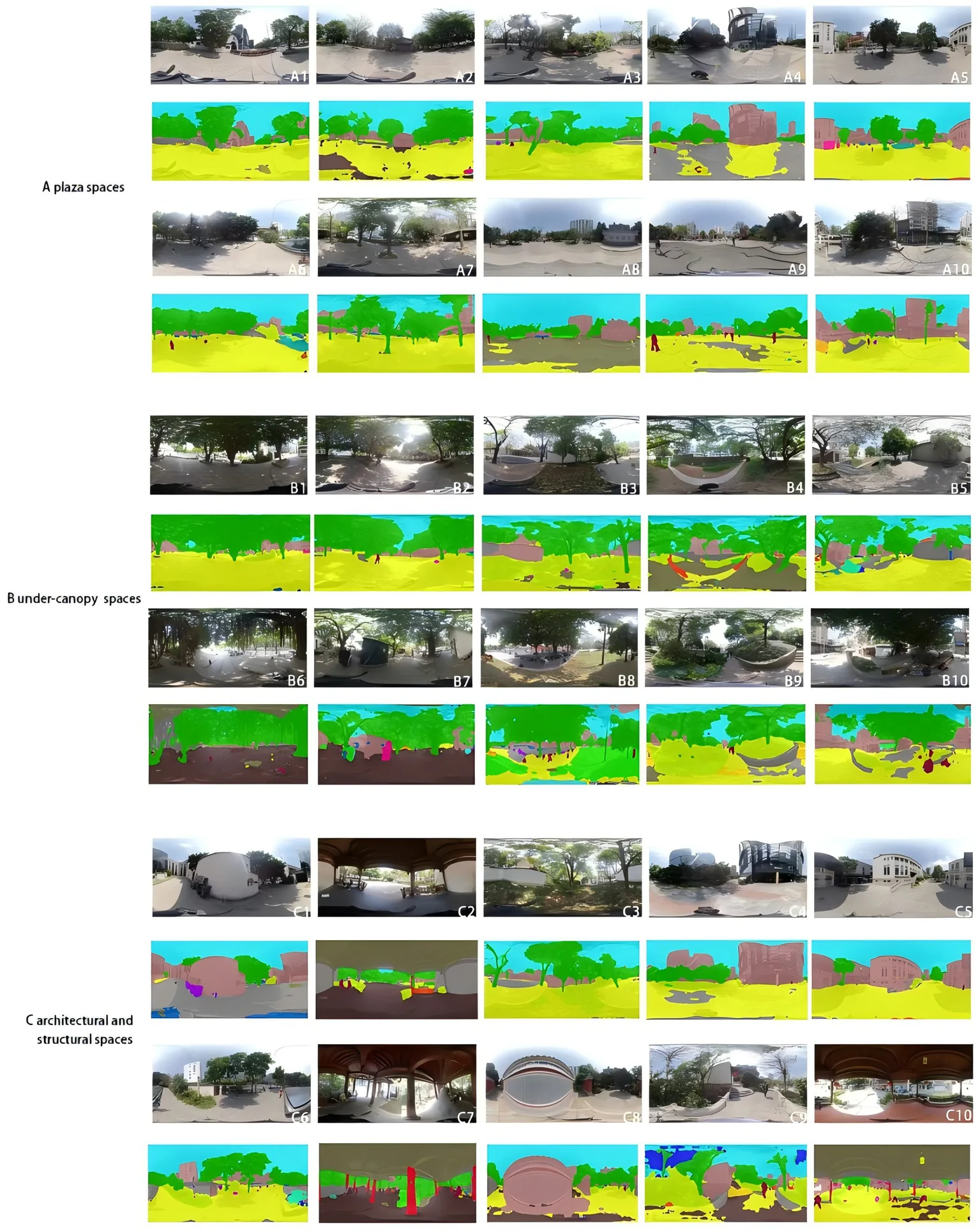
Semantic segmentation results of the 30 panoramic images.

A panoramic image was captured at each of the 30 observation points using an Insta360 ONE X camera mounted on a tripod 2.5–3.0 m above ground level. Semantic segmentation of the panoramic images was performed using the DeepLab-V3 + neural network algorithm proposed by the Google Research team in 2018. By integrating local spectral information at the pixel level with global contextual information, the DeepLab-V3 + algorithm improves image feature extraction. Segmentation outputs from 10 of the 30 observation points were compared with manual visual interpretation, yielding an accuracy of 82.1%. The 11 visual landscape elements were identified and segmented in the panoramic images using the ADE20K dataset. The segmentation results were then used to calculate the proportion of each landscape element at each of the 30 observation points (Figure 4).

#### 2.2.2 Physiological and psychological measures

##### 2.2.2.1 Physiological responses

**Figure 5:**
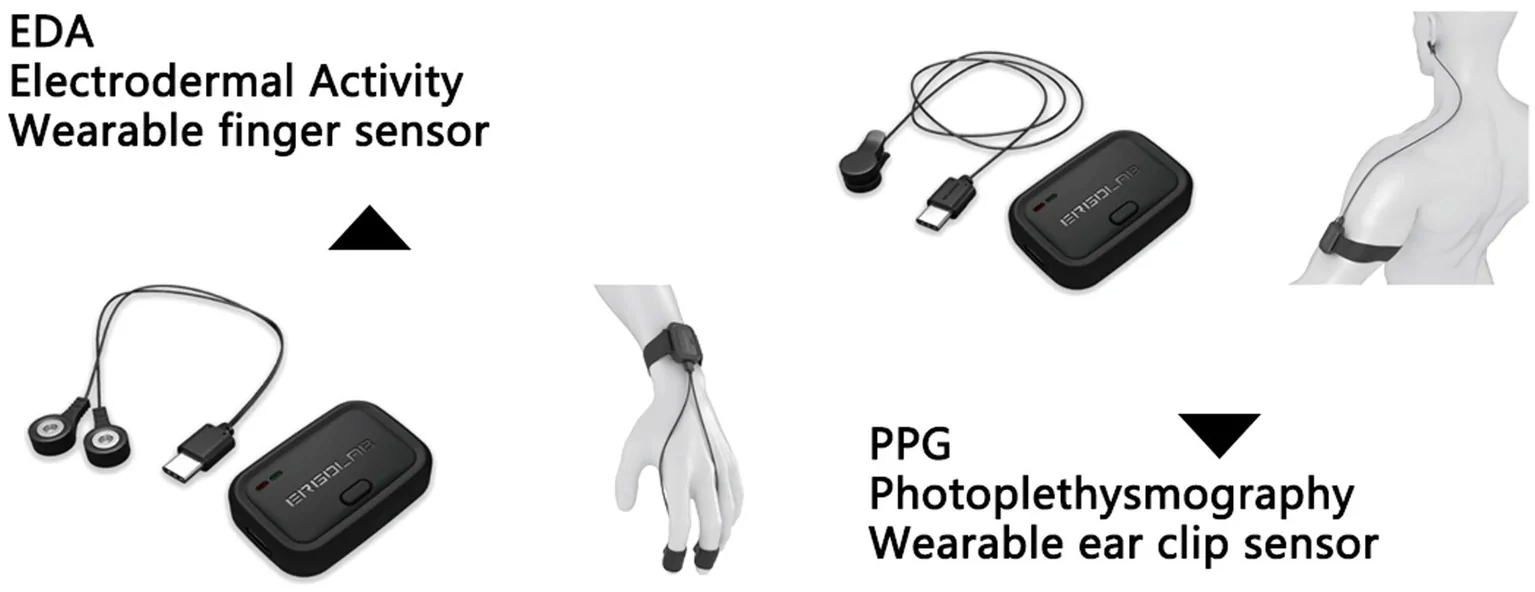
ErgoLAB intelligent wearable ergonomics platform.

The ErgoLAB intelligent wearable ergonomics platform (Beijing Jinfa Technology Co., Ltd., Beijing, China) was used to record participants’ photoplethysmography (PPG) and electrodermal activity (EDA) signals. Data were sampled at 64 Hz and transmitted wirelessly to a computer for real-time recording and analysis (Figure 5). In this study, skin conductance level (SCL) and heart rate variability (HRV) indices were selected to characterize short-term physiological responses to pocket park environments. The physiological outcomes comprised skin conductance level (SCL) and six cardiac measures: interbeat interval (IBI), heart rate (HR), standard deviation of normal-to-normal intervals (SDNN), root mean square of successive differences (RMSSD), the percentage of successive normal-to-normal intervals differing by more than 50 ms (pNN50), and the ratio of low-frequency to high-frequency power (LF/HF). These indicators have been widely used in studies of environmental psychology, restorative environments, and urban green spaces to evaluate physiological responses associated with environmental exposure and restoration-related experiences. Specifically, HR reflects changes in cardiovascular activation, pNN50 is commonly used to characterize parasympathetic-related cardiac activity, LF/HF has frequently been employed as an indicator associated with autonomic regulation, and SCL reflects physiological arousal associated with sympathetic nervous system activation. The physiological significance of all indicators is summarized in Table 1 (28). Considering that physiological indicators may be influenced by multiple individual and environmental factors, the observed changes should be interpreted with appropriate caution, particularly for LF/HF, whose physiological interpretation may be affected by factors such as respiration and autonomic activity (29, 30).

**Table 1:** Physiological indicators and their interpretation.

Indicator	Definition	Physiological interpretation
SCL/μS	Skin conductance level, the absolute value of skin electricity between two points on the skin	Reflects sympathetic nerve activity and emotional arousal.
IBI/ms	Interval between adjacent normal R waves	Reflects heart rate variability, related to blood pressure.
HR/bpm	Heart rate, number of heartbeats per minute in a resting state	Reflects individual physiological state, arousal, and stress levels.
SDNN/ms	Standard deviation of normal R-R interval	Reflects slow components of heart rate variability, related to parasympathetic and sympathetic activation. Higher values indicate higher HRV.
RMSSD/ms	Root mean square of successive R-R interval differences	Indicates parasympathetic activity, with increased parasympathetic activity leading to higher RMSSD.
pNN50/%	Percentage of adjacent RR intervals with a difference >50 ms	Reflects sudden changes in R-R intervals, providing a more timely measure of parasympathetic activity.
LF/HF	Ratio of low frequency to high frequency in HRV	Has been widely used as an indirect indicator of autonomic regulation; however, its physiological interpretation remains debated and should be interpreted with caution.

##### 2.2.2.2 Psychological responses

Psychological responses were assessed using the Perceived Restorativeness Scale (PRS) and the Environmental Preference Scale. The adapted PRS comprised 18 items across four dimensions: Being Away, Extent, Fascination, and Compatibility. The Environmental Preference Scale comprised 14 items covering four dimensions: Coherence, Legibility, Complexity, and Mystery (31, 32). All items were rated on a seven-point Likert scale ranging from 1 (strongly disagree) to 7 (strongly agree).

#### 2.2.3 Participants and experimental procedures

An *a priori* sample-size calculation was conducted using G*Power 3.1.9.2. With an effect size of *f* = 0.25, α = 0.05, and statistical power of 0.80, the calculation indicated a minimum sample of 27 participants (33). Previous studies have demonstrated that university students exhibit stable cognitive and physiological responses to environmental stimuli and are suitable participants for environmental perception and restorative environment research (34–36). Accordingly, a total of fifty university students with no formal training in landscape architecture were recruited on campus for the experiment. The sample comprised 22 males and 28 females, with a mean age of 24.6 ± 3.2 years.

The inclusion criteria were as follows: (1) participants were physically healthy and had no history of cardiovascular disease; (2) participants had not previously taken part in similar experiments; (3) participants provided written informed consent prior to participation. Each participant received compensation of CNY 50 upon completion of the experiment.

**Figure 6:**
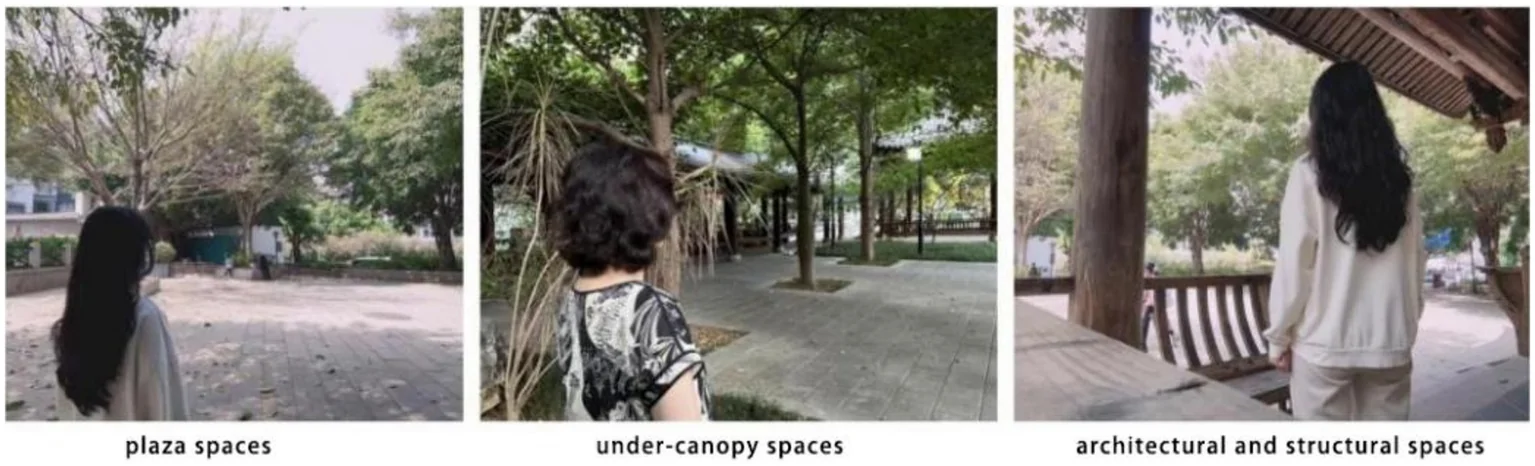
Field observations in the three visual-setting categories.

The experiment was conducted between 6 May and 3 June 2024 during two daily periods (08:00–11:00 and 14:00–17:00). Ambient temperatures at the observation sites ranged from 25 °C to 29 °C, a range considered thermally comfortable for participants. The experimental procedure consisted of the following steps:

1. Upon arrival at the laboratory, participants rested quietly for five minutes to allow their heart rate and respiration to stabilize. During this period, participants were instructed to remain seated, avoid vigorous movement, and refrain from using electronic devices.
2. Participants were then fitted with the physiological monitoring equipment and received standardized instructions regarding the experimental procedure and use of the equipment. Baseline physiological data, including electrodermal activity (EDA) and photoplethysmography (PPG), were recorded continuously for three minutes. During baseline acquisition, participants remained relaxed, awake, and silent.
3. After being transported to the study sites, participants visited the designated observation points according to a standardized observation schedule. Participants moved between observation points at a normal walking pace under the guidance of the research team. To minimize the influence of physical activity on physiological measurements, physiological data were analyzed only during the stationary observation periods. At each observation point, participants remained standing quietly while viewing the surrounding landscape for five minutes and were instructed to avoid unnecessary movement, conversation, and mobile phone use. Physiological signals were recorded continuously throughout the observation period.
4. Upon completion of the landscape observation sessions, the physiological monitoring equipment was removed and participants completed the psychological response questionnaires, including the Perceived Restorativeness Scale and Environmental Preference Scale. All participants followed the same experimental protocol throughout the study. Representative photographs of the field observations in the three recreational space types are shown in Figure 6.

Because the experiment was conducted under real-world field conditions, complete randomization of observation sequences across all sites was not feasible. Nevertheless, observation schedules were organized in advance to minimize potential order effects and participant fatigue. Therefore, the findings should be interpreted primarily as associations between visual landscape characteristics and psychological and physiological responses under field conditions.

### 2.3 Data processing and statistical analysis

Physiological signals were processed in ErgoLAB to derive the cardiac and electrodermal measures used in the analysis. Statistical analyses were performed in SPSS 23.0 (IBM Corp., Armonk, NY, USA). Multicollinearity among the visual landscape indicators was assessed using variance inflation factors (VIFs). Indicators with a VIF greater than 5 were excluded before model estimation; this procedure resulted in the removal of the green view index.

Separate multiple linear regression models were fitted as the primary analysis for the four dimensions of environmental preference, the four dimensions of perceived restorativeness, and the seven physiological outcomes. Standardized regression coefficients (β) and *p* values were reported, with *p* < 0.05 used as the threshold for statistical significance. These models were used to examine the associations between visual landscape characteristics and psychological and physiological responses. Supplementary stratified analyses were additionally conducted for plaza, under-canopy, and architectural/structural spaces to explore whether the observed associations varied across different spatial contexts.

Because the dataset included repeated observations, linear mixed-effects models (LMMs) were conducted as a supplementary sensitivity analysis. Visual landscape indicators were specified as fixed effects, with observation point/scene as a random intercept to account for scene-level clustering. The same variables as in the primary multiple linear regression analyses were examined to assess the robustness of the observed associations.

## 3 Results

### 3.1 Associations between visual landscape characteristics and environmental preference

All four regression models were statistically significant (all *p* < 0.001; Table 2). Tree proportion was positively associated with Coherence, Legibility, Complexity, and Mystery (*β* = 0.298—0.484, *p* ≤ 0.003), showing the most consistent association across the four dimensions. Lawn proportion was positively associated with Legibility (*β* = 0.111, *p* = 0.027), while shrub proportion was positively associated with Complexity and Mystery (*β* = 0.112 and 0.170, respectively; *p* ≤ 0.048). Distant mountain visibility was positively associated with Complexity and Mystery (*β* = 0.156 and 0.174; *p* ≤ 0.045). In contrast, hard pavement was negatively associated with Legibility and Complexity (*β* = −0.236 and −0.218; both *p* = 0.002). Building proportion was negatively associated with Complexity and Mystery (*β* = −0.152 and −0.187, respectively; *p* ≤ 0.025), while fence proportion was negatively associated with Mystery (*β* = −0.162, *p* = 0.045). To further examine whether these associations varied across recreational space types, a supplementary stratified analysis was conducted for plaza, under-canopy, and architectural/structural spaces. In plaza spaces, tree and building proportions were negatively associated with Legibility (*β* = −1.029 and −1.002, respectively; *p* = 0.034 and 0.032). In architectural/structural spaces, shrub proportion showed a strong positive association with Mystery (*β* = 0.967, *p* = 0.006), and the corresponding model explained a relatively high proportion of variance (R^2^ = 0.871; adjusted R^2^ = 0.768; *p* = 0.019). These findings complement the pooled analysis by suggesting that the relationships between visual landscape characteristics and environmental preference may vary across spatial contexts, with vegetation-related characteristics appearing particularly relevant in architectural/structural spaces.

**Table 2:** Multiple linear regression results for the four dimensions of environmental preference.

Predictor variables		Model 1		Model 2		Model 3		Model 4	
		Standardized Coefficient (*β*)	*P*	Standardized Coefficient (*β*)	*P*	Standardized Coefficient (*β*)	*P*	Standardized Coefficient (*β*)	*P*
	Constant	——	0.000^**^	——	0.000^**^	——	0.000^**^	——	0.002^**^
Plant landscape elements	Proportion of trees	0.298	0.003^**^	0.352	0.000^**^	0.361	0.000^**^	0.484	0.000^**^
	Proportion of shrubs	0.088	0.143	−0.003	0.959	0.112	0.048^*^	0.170	0.003^**^
	Proportion of lawn	0.041	0.424	0.111	0.027^*^	0.042	0.382	0.052	0.277
Architectural and built landscape elements	Proportion of structures	−0.013	0.809	0.026	0.626	0.046	0.375	0.076	0.137
	Proportion of buildings	−0.054	0.452	−0.086	0.226	−0.152	0.025^*^	−0.187	0.006^**^
	Proportion of hard pavement	0.015	0.847	−0.236	0.002^**^	−0.218	0.002^**^	0.087	0.216
	Proportion of fences	0.11	0.203	−0.001	0.986	−0.106	0.195	−0.162	0.045^*^
Spatial landscape elements	Proportion of visible sky	0.017	0.841	0.095	0.267	0.152	0.063	0.122	0.131
	Proportion of distant Mountains	−0.083	0.313	0.061	0.451	0.156	0.045^*^	0.174	0.024^*^
	Spatial enclosure	−0.176	0.090	−0.04	0.692	0.692	0.276	−0.039	0.683
Dependent variables		Coherence		Legibility		Complexity		Mystery	
Model evaluation		*p* = 0.000^**^		*P* = 0.000^**^		*P* = 0.000^**^		*P* = 0.000^**^	

*p < 0.05;**p < 0.01.

**Figure 7:**
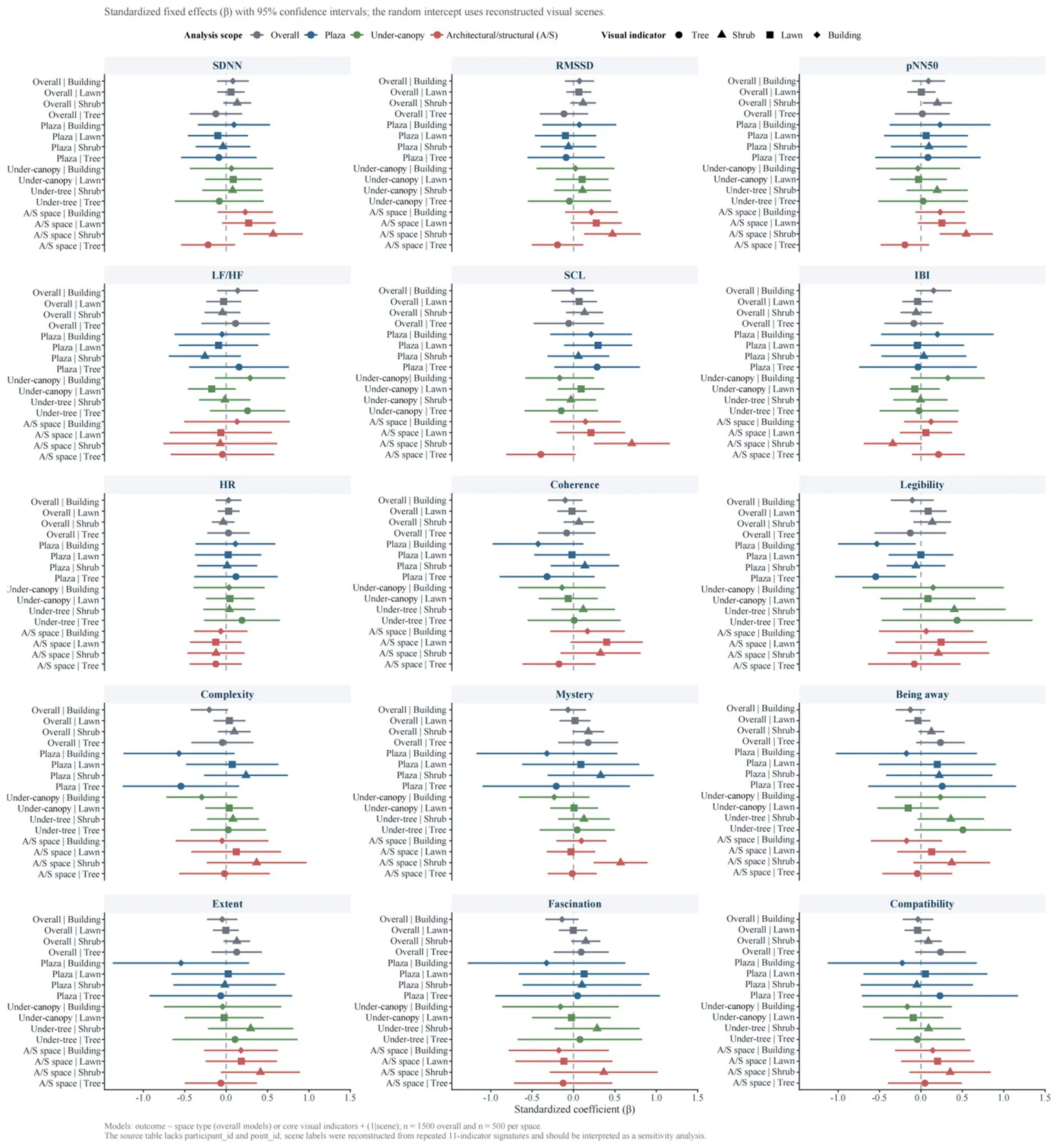
Mixed-effects associations between visual elements and all outcomes.

As a supplementary sensitivity analysis, linear mixed-effects models were additionally conducted to account for the repeated-measures structure of the observations. Although the overall associations were attenuated compared with the primary regression analysis, several context-specific patterns remained evident. In plaza spaces, tree and building proportions remained negatively associated with Legibility at the nominal significance level (*β* = −0.546 and −0.532, respectively; *p* = 0.034 and 0.032). More notably, in architectural/structural spaces, shrub proportion remained positively associated with mystery (*β* = 0.569, *p* = 0.006), and this association remained significant after adjustment for multiple comparisons (*q* = 0.039). These findings provide supplementary support for the context-dependent relationship between visual landscape characteristics and environmental preference, particularly highlighting the potential relevance of shrub-related characteristics in architectural/structural spaces (Figure 7).

### 3.2 Associations between visual landscape characteristics and perceived Restorativeness

All four regression models were statistically significant (all *p* < 0.001; Table 3). Tree proportion was positively associated with Being Away, Extent, Fascination, and Compatibility (*β* = 0.500–0.570, all *p* < 0.001). Shrub proportion and distant mountain visibility were also positively associated with all four dimensions (shrub: *β* = 0.152–0.259, *p* ≤ 0.007; distant mountains: *β* = 0.223–0.323, *p* ≤ 0.004). Lawn proportion was positively associated with Extent and Compatibility (*β* = 0.087 and 0.129; *p* = 0.045 and 0.007, respectively). In contrast, fence proportion and spatial enclosure were negatively associated with all four dimensions (fences: *β* = −0.151 to −0.268, *p* ≤ 0.040; spatial enclosure: *β* = −0.190 to −0.399, *p* ≤ 0.039), while building proportion was negatively associated with Extent, Fascination, and Compatibility (*β* = −0.128 to −0.226, *p* ≤ 0.037). A supplementary stratified analysis was conducted to examine whether these associations varied across plaza, under-canopy, and architectural/structural spaces. After aggregation of repeated observations at the scene level, none of the four perceived-restorativeness models in the plaza or under-canopy spaces reached statistical significance. Similarly, in architectural/structural spaces, the overall models for Being Away, Extent, Fascination, and Compatibility were not statistically significant, with *p* values of 0.157, 0.367, 0.376, and 0.396, respectively. Nevertheless, at the coefficient level, shrub proportion showed positive associations with several dimensions of perceived restorativeness in architectural/structural spaces. This pattern was broadly consistent with the trend observed in the pooled analysis.

**Table 3:** Multiple linear regression results for the four dimensions of perceived restorativeness.

Predictor variables		Model 1		Model 2		Model 3		Model 4	
		Standardized coefficient (*β*)	*P*	Standardized coefficient (*β*)	*P*	Standardized coefficient (*β*)	*P*	Standardized coefficient (*β*)	*P*
	Constant	——	0.000^**^	——	0.000^**^	——	0.000^**^	——	0.000^**^
Plant landscape elements	Proportion of trees	0.500	0.000^**^	0.529	0.000^**^	0.529	0.000^**^	0.570	0.000^**^
	Proportion of shrubs	0.259	0.000^**^	0.177	0.001^**^	0.180	0.001^**^	0.152	0.007^**^
	Proportion of lawn	0.051	0.248	0.087	0.045^*^	0.069	0.125	0.129	0.007^**^
Architectural and built landscape elements	Proportion of structures	−0.079	0.093	−0.040	0.394	0.046	0.342	−0.031	0.537
	Proportion of buildings	−0.100	0.108	−0.128	0.037^*^	−0.226	0.000^**^	−0.141	0.035^*^
	Proportion of hard pavement	0.097	0.136	0.023	0.721	0.007	0.919	0.110	0.117
	Proportion of fences	−0.192	0.010^*^	−0.151	0.040^*^	−0.268	0.001^**^	−0.245	0.002^**^
Spatial landscape elements	Proportion of visible sky	−0.038	0.610	−0.008	0.915	0.066	0.393	0.030	0.713
	Proportion of distant Mountains	0.323	0.000^**^	0.238	0.001^**^	0.301	0.000^**^	0.223	0.004^**^
	Spatial enclosure	−0.399	0.000^**^	−0.333	0.000^**^	−0.190	0.039^*^	−0.265	0.006^**^
Dependent variables		Being away		Extent		Fascination		Compatibility	
Model evaluation		*p* = 0.000^**^		*P* = 0.000^**^		*P* = 0.000^**^		*P* = 0.000^**^	

*p < 0.05;**p < 0.01.

As a supplementary sensitivity analysis, linear mixed-effects models were additionally conducted to account for the repeated-measures structure. The results indicated that associations between visual landscape characteristics and perceived restorativeness remained evident for selected dimensions. The overall model for Being Away remained statistically significant (*p* = 0.004), while the model for Fascination also reached nominal statistical significance (*p* = 0.041). Although the overall models for Extent and Compatibility did not reach conventional statistical significance (*p* = 0.153 and 0.105, respectively), the estimated effects of tree proportion on the four dimensions remained consistently positive, particularly for Being Away (*β* = 0.236) and Compatibility (*β* = 0.237). Taken together, these supplementary findings suggest that the associations between visual landscape characteristics and perceived restorativeness were attenuated to some extent after accounting for repeated observations, but that meaningful relationships remained evident for selected restorative dimensions. The results therefore provide additional support for the potential role of visual landscape characteristics in shaping restorative environmental experiences, while also suggesting that these relationships may vary according to spatial context (Figure 7).

### 3.3 Associations between visual landscape characteristics and physiological outcomes

Four of the seven physiological models were statistically significant: HR (*p* = 0.003), pNN50 (*p* = 0.011), LF/HF (*p* = 0.005), and SCL (*p* = 0.005), whereas the models for IBI, SDNN, and RMSSD did not reach statistical significance (*p* = 0.056, 0.125, and 0.172, respectively; Table 4). In the HR model, tree, shrub, lawn, and distant mountain proportions were negatively associated with heart rate (*β* = −0.105 to −0.196, *p* ≤ 0.007), whereas building proportion, fence proportion, and spatial enclosure were positively associated with heart rate (*β* = 0.158–0.179, *p* ≤ 0.035). Fence proportion was negatively associated with pNN50 (*β* = −0.222, *p* = 0.018). Tree proportion and visible sky proportion were negatively associated with LF/HF (*β* = −0.241 and −0.221; *p* = 0.023 and 0.019, respectively), while hard pavement was positively associated with LF/HF (*β* = 0.229, *p* = 0.005). Hard pavement was also positively associated with SCL (*β* = 0.190, *p* = 0.019), whereas lawn proportion was negatively associated with SCL (*β* = −0.123, *p* = 0.025). The supplementary stratified analysis further indicated that the associations between visual landscape characteristics and physiological responses varied across recreational space types. The most pronounced model-level associations were observed in architectural/structural spaces, where the overall models for pNN50 (R^2^ = 0.826; adjusted R^2^ = 0.687; *p* = 0.039) and HR (R^2^ = 0.827; adjusted R^2^ = 0.688; *p* = 0.038) were statistically significant. At the coefficient level, shrub proportion was positively associated with SDNN (*β* = 1.147), RMSSD (*β* = 1.087), pNN50 (*β* = 1.183), and SCL (*β* = 1.115). These findings suggest that vegetation-related characteristics may be associated with physiological responses in architectural/structural spaces. However, given the small number of independent scenes within each spatial type, these stratified findings should be interpreted cautiously as supplementary, exploratory findings.

**Table 4:** Multiple linear regression results for the physiological outcomes.

Predictor variables		Model 1		Model 2		Model 3		Model 4		Model 5		Model 6		Model 7	
		Standardized Coefficient (*β*)	*P*	Standardized Coefficient (*β*)	*P*	Standardized Coefficient (*β*)	*P*	Standardized Coefficient (*β*)	*P*	Standardized Coefficient (*β*)	*P*	Standardized Coefficient (*β*)	*P*	Standardized Coefficient (*β*)	*P*
	Constant	——	0.409	——	0.000^**^	——	0.189	——	0.306	——	0.968	——	0.011^*^	——	0.609
Plant landscape elements	Proportion of trees	0.124	0.244	−0.196	0.000^**^	0.000	0.997	0.012	0.912	0.144	0.174	−0.241	0.023^*^	0.194	0.067
	Proportion of shrubs	−0.013	0.844	−0.172	0.007^**^	0.061	0.350	0.050	0.447	0.046	0.472	−0.115	0.073	0.002	0.969
	Proportion of lawn	−0.023	0.670	−0.129	0.007^**^	−0.021	0.704	−0.014	0.798	−0.074	0.179	−0.056	0.307	−0.123	0.025^*^
Architectural and built landscape elements	Proportion of structures	−0.076	0.200	0.080	0.537	−0.037	0.536	−0.054	0.367	−0.007	0.906	0.017	0.768	0.110	0.062
	Proportion of buildings	0.091	0.241	0.179	0.035^*^	0.086	0.274	0.112	0.156	0.081	0.298	0.016	0.840	−0.089	0.251
	Proportion of hard pavement	−0.009	0.913	0.020	0.117	−0.031	0.710	0.003	0.969	−0.004	0.957	0.229	0.005^**^	0.190	0.019^*^
	Proportion of fences	0.126	0.180	0.158	0.002^**^	0.239	0.012^*^	0.237	0.013^*^	−0.222	0.018^*^	0.071	0.447	0.116	0.214
Spatial landscape elements	Proportion of visible sky	−0.044	0.637	−0.029	0.713	−0.064	0.499	−0.019	0.839	−0.063	0.501	−0.221	0.019^*^	0.001	0.988
	Proportion of distant mountains	−0.092	0.304	−0.105	0.004^**^	−0.139	0.123	−0.140	0.121	−0.142	0.110	−0.035	0.695	−0.023	0.791
	Spatial enclosure	−0.135	0.229	0.170	0.006^**^	0.010	0.932	−0.004	0.973	0.079	0.481	−0.031	0.782	0.111	0.317
Dependent variables		IBI		HR		SDNN		RMSSD		pNN50		LF/HF		SCL	
Model evaluation		*p* = 0.056		*p* = 0.003^**^		*p* = 0.125		*p* = 0.172		*p* = 0.011^*^		*p* = 0.005^**^		*P* = 0.005^**^	

*p < 0.05;**p < 0.01.

As a supplementary sensitivity analysis, linear mixed-effects models were additionally conducted to account for the repeated-measures structure. Although none of the seven overall physiological models reached conventional statistical significance, several consistent context-specific associations were observed in architectural/structural spaces. In particular, shrub proportion was positively associated with SDNN (*β* = 0.568, *p* = 0.009, *q* = 0.039), RMSSD (*β* = 0.470, *p* = 0.015, *q* = 0.046), pNN50 (*β* = 0.547, *p* = 0.007, *q* = 0.039), and SCL (*β* = 0.705, *p* = 0.010, *q* = 0.039), with these associations remaining significant after adjustment for multiple comparisons. These supplementary findings are broadly consistent with the context-specific pattern identified in the primary analyses and further highlight the potential relevance of shrub-related visual characteristics to physiological responses in architectural/structural spaces. Taken together, the results suggest that physiological responses to visual landscape characteristics may be context-dependent rather than uniform across recreational space types. Given the limited number of independent scenes within each spatial category, these findings should be interpreted as exploratory and warrant further investigation in studies involving a larger number of independent scenes (Figure 7).

## 4 Conclusions and limitations

### 4.1 Conclusion

1. The findings indicate that participants’ environmental preference and perceived restorativeness were significantly associated with several visual landscape characteristics of pocket parks. In the primary regression analyses, higher proportions of trees, shrubs, lawns, and distant mountains were generally associated with higher environmental preference and perceived restorativeness, whereas higher proportions of buildings, hard-paved surfaces, fences, and greater spatial enclosure were generally associated with lower psychological evaluations (*P* < 0.05). Overall, participants tended to report more favorable environmental evaluations in landscape settings characterized by greater vegetation visibility, richer vegetation composition, and greater visual openness. The supplementary stratified analyses further suggested that these associations varied across recreational space types. In particular, shrub proportion showed positive associations with environmental preference in architectural/structural spaces, while tree and building proportions were associated with Legibility in plaza spaces. The supplementary LMM analyses further indicated that some of these context-specific associations remained evident after accounting for the repeated-measures structure, particularly the positive association between shrub proportion and Mystery in architectural/structural spaces. Taken together, these findings suggest that the relationship between visual landscape characteristics and environmental preference and perceived restorativeness is context-dependent rather than uniform across recreational space types. From a design perspective, maintaining appropriate vegetation visibility and spatial openness may therefore be considered when developing different types of pocket park recreational spaces.
2. Visual landscape characteristics were also associated with physiological responses in the primary regression analyses. Vegetation-related indicators, particularly tree, shrub, and lawn proportions, were generally associated with lower heart rate and more favorable patterns in selected autonomic and electrodermal indicators (*P* < 0.05), whereas higher proportions of buildings, hard-paved surfaces, and fences were generally associated with greater physiological arousal. Greater visible-sky proportion and distant-mountain visibility were also associated with more favorable short-term physiological responses, while greater spatial enclosure was generally associated with less favorable physiological patterns. The supplementary stratified analyses indicated that the strength and pattern of these associations varied across recreational space types, with the most pronounced model-level associations observed in architectural/structural spaces. In these spaces, shrub proportion was positively associated with SDNN, RMSSD, pNN50, and SCL. Importantly, the supplementary LMM analyses identified consistent positive associations between shrub proportion and these physiological indicators in architectural/structural spaces, which remained significant after multiple-comparison adjustment. Taken together, the primary and supplementary analyses suggest that the relationship between visual landscape characteristics and physiological responses may be dependent on spatial context, with vegetation-related characteristics potentially playing a particularly important role in more enclosed architectural/structural settings. From a design perspective, increasing green visibility, reducing the visual dominance of hardscape elements, and maintaining appropriate levels of spatial openness may be considered in health-supportive and restoration-oriented pocket park design, while taking into account the distinct characteristics of different recreational space types.

### 4.2 Limitations

1. The participant sample consisted exclusively of university students. Although university students are frequently used in environmental perception and restorative environment research because of their relatively stable cognitive and physiological responses and high adherence to experimental procedures, the sample may not fully represent the diversity of urban park users. Therefore, caution should be exercised when generalizing the findings to populations from different age groups, socioeconomic backgrounds, health conditions, or recreational preferences.
2. All study sites were located in Fuzhou, a city characterized by a humid subtropical climate, abundant vegetation, and distinct urban-development characteristics. Consequently, the findings may not be directly transferable to cities with substantially different climatic, cultural, or spatial contexts. Nevertheless, several observed associations, particularly those related to vegetation visibility, spatial openness, and the visual dominance of built structures, are generally consistent with findings reported in previous studies on urban green spaces and restorative environments. This suggests that some of the identified relationships may have broader relevance in high-density urban settings. However, the magnitude and relative importance of specific visual landscape characteristics may vary according to local environmental conditions, vegetation composition, cultural preferences, and patterns of park use. Future comparative studies across multiple cities are needed to further evaluate the transferability and robustness of these findings.
3. The present study focused on short-term psychological and physiological responses during exposure to pocket park environments. Therefore, the findings should be interpreted primarily as associations between visual landscape characteristics and short-term restoration-related responses rather than evidence of long-term health outcomes or public health effects.

### 4.3 Future research directions

Future studies should expand the geographical scope of investigation to include pocket parks located in different climatic, cultural, and socioeconomic contexts. Comparative studies across multiple cities and regions would help evaluate the transferability and robustness of the observed associations between visual landscape characteristics and psychological and physiological responses.

In addition, landscape experiences are inherently multisensory. Beyond visual perception, future research should further investigate the interactive effects of auditory, olfactory, tactile, and other sensory stimuli on psychological and physiological responses. Exploring the mechanisms of multisensory integration may provide a more comprehensive understanding of human-environment interactions and support the development of evidence-based landscape design strategies.

## Data Availability

The raw data supporting the conclusions of this article will be made available by the authors, without undue reservation.
